# CYP450 Genotype—Phenotype Concordance Using the Geneva Micrococktail in a Clinical Setting

**DOI:** 10.3389/fphar.2021.730637

**Published:** 2021-08-26

**Authors:** Kuntheavy Ing Lorenzini, Jules Desmeules, Victoria Rollason, Stéphane Bertin, Marie Besson, Youssef Daali, Caroline F. Samer

**Affiliations:** Division of Clinical Pharmacology and Toxicology, Department of Anaesthesiology, Pharmacology, Intensive Care and Emergency Medicine, University Hospitals of Geneva, Geneva, Switzerland

**Keywords:** cytochromes P450, phenotype, genotype, phenoconversion, drug-drug interaction, inefficacy, adverse drug reaction

## Abstract

Pharmacokinetic variability is a major source of differences in drug response and can be due to genetic variants and/or drug-drug interactions. Cytochromes P450 are among the most studied enzymes from a pharmacokinetic point of view. Their activity can be measured by phenotyping, and/or predicted by genotyping. Depending on the presence of drugs and/or diseases that can affect their *in vivo* activity, both approaches can be complementary. In 2014, the Geneva cocktail using dried blood spots was validated in healthy volunteers for CYP450 phenotyping. Since its clinical implementation, it has been used in approximately 500 patients in various clinical situations. Our study aims to report the concordance between CYP450 genotype and phenotype in real-life patients. The prospectively collected data from patients who were genotyped and/or phenotyped between January 2014 and December 2020 were reviewed. A total of 537 patients were genotyped and/or phenotyped for CYP450 during this period, and 241 underwent simultaneous genotyping and phenotyping allowing for genotype/phenotype concordance assessment. Genotyping correctly predicted poor metabolizer phenotypes for most CYPs isoenzymes studied, whereas agreement was more variable for intermediate, normal, and ultrarapid metabolizers. Discrepancies between the phenotype predicted on the basis of genotyping and the measured phenotype were not always explained by concurrent medication (phenotypic switch). Therefore genotyping and phenotyping tests are complementary approaches when aiming to individualize drug therapy. In the 537 patients, the majority of clinical situations were observed with analgesic/anesthetic drugs (*n* = 187), followed by antidepressants (*n* = 153), antineoplastics (*n* = 97), and immunosuppressants (*n* = 93). Inefficacy (or low drug levels) and adverse drug reaction (or high drug levels) were the main reasons for testing. Genotype and/or phenotype results explained or at least contributed to the clinical event in 44% of cases.

## Introduction

Patients vary in their response to drugs. A dose that is effective in a given patient may cause an adverse drug reaction (ADR) in another patient or conversely be ineffective. Several causes of variability can be cited, genetic- or disease-related changes in drug concentrations or responsiveness, poor compliance, drug-drug interactions (DDI). Variability in drug response can affect pharmacokinetics, pharmacodynamics, or both (Roden et al., 2019). Pharmacokinetic variability is a major source of differences in drug response and can be due to genetic variants, diseases themselves and/or DDI. Cytochromes P450 (CYP450) are among the most studied enzymes from a pharmacokinetic point of view. Their activity can be predicted by genotyping and/or measured by phenotyping.

Genotyping consists of determining the patient DNA sequence and analyzing functional genetic variants coding for specific enzymes. It allows predicting the phenotype based on the identified alleles (Samer et al., 2013). The genotype offers the advantage of being immutable in a given patient. However, predicting metabolic phenotype from genotype may be challenging for CYP450 enzymes, especially given the continuously increasing number of novel alleles being discovered (Shah et al., 2016). In practice, the genotype does not necessarily correlate well with the phenotype (Waring, 2020). Another major issue is that genotyping does not account for any of the many environmental factors (diseases, drug interactions, dietary) which may impact phenotype (McGraw et al., 2018).

On the other hand, phenotyping can be considered a more useful tool for patient metabolism evaluation to anticipate possible inefficacy or ADR at conventional doses (Keller et al., 2017). However, it implies the oral administration of probe specific xenobiotics, followed by blood or urine sampling. This may represent a limitation in vulnerable populations such as children and pregnant women. Therefore, we believe that genotyping and phenotyping are complementary in clinical settings, depending on the presence of drugs and/or diseases that may affect the *in vivo* activity of CYP450.

At Geneva University Hospitals, we have been using, CYP450 genotyping and phenotyping methods in patients for almost two decades. *In vivo* phenotyping can be performed by administering a single probe drug metabolized by an individual CYP enzyme, or by a “cocktail” approach, consisting in administering several probe drugs. The cocktail approach allows for the simultaneous determination of several CYP enzymes activity (Keller et al., 2017). The first Geneva phenotyping cocktail for CYP450 phenotyping was developed in 2004. It contained 5 probe substrates used at therapeutic doses (100 mg caffeine, 50 mg flurbiprofen, 40 omeprazole, 25 mg dextromethorphan, and 7.5 mg midazolam), thus associated with a risk of therapeutic unwanted effects (Jerdi et al., 2004). Our clinical experience in patients using the “full dose” phenotyping cocktail and its subsequent variants has been published previously with psychotropic drugs (Lloret-Linares et al., 2016) and analgesic drugs (Rollason et al., 2020a). In 2014, a new mixed-probe of the Geneva cocktail, which is called the Geneva microcoktail, using dried blood spots, was validated in healthy volunteers. The Geneva micrococktail contains smaller doses of probe substrates: 50 mg caffeine, 20 mg bupropion, 10 mg flurbiprofen, 10 omeprazole, 10 mg dextromethorphan, 1 mg midazolam, and 25 mg fexofenadine (Bosilkovska et al., 2014), facilitates sample collection, requiring only 10 µL blood samples, and allows phenotyping for additional CYPs and P-glycoprotein. This new formulation of the Geneva cocktail recently showed an excellent safety profile in 265 healthy volunteers from different geographic regions (Rollason et al., 2020b). It was also used to characterize the variation in CYP450 activity in patients undergoing elective orthopedic surgery (Lenoir et al., 2020).

The clinical use of the Geneva micrococktail in real-life polymorbid and/or polymedicated patients has not been previously reported. Since its implementation, we have used it in approximately 500 patients. Our study aims to report the concordance between CYP450 genotype and phenotype using the Geneva micrococktail in real-life patients. Second, we aim to determine if genotyping and/or phenotyping help explaining unexpected clinical responses to drug administration (ADR, or inefficacy).

## Methods

### Patients and Setting

The protocol of this retrospective study was approved by the Ethics Commission of the Canton of Geneva, Geneva (Switzerland) (study number: 15-225). We evaluated in- and outpatients with CYP450 genotyping and/or phenotyping tests since 2014, the year of implementation of the Geneva micrococktail as used in its current formulation. As described previously (Lloret-Linares et al., 2016; Rollason et al., 2020a), we retrospectively collected results of the genetic and/or phenotypic investigations made because of non-response to drugs or excessive response to drugs performed between April 2014 and December 2020. Our previously published worked included patients who underwent phenotyping until November 2014. Since we included patients from April 2014, there is an overlap of 18 patients included in the previous as well as in the present article. In the present paper, we will focus on genotype-phenotype concordance. Therefore, patients with genotype-only testing will not be discussed in detail unlike our previously published articles. Patients with only non-CYP450 genotyping (e.g. ABCB1, COMT) were excluded from the analysis.

### Genotyping

Genotyping was performed by our institutional laboratory of molecular oncology and pharmacogenomics. Genotyping techniques have considerably evolved over the last few years. From 2017, the used method was based on Next Generation Sequencing (NGS) technologies with the pharmacogenomics panel from ThermoFisher. CYP2D6 gene copy number was determined by qPCR on LC480 (Roche) using CNV Exon 9 Hs00010001_cn and CNV Intron 6 Hs04502391_cn probes for CYP2D6 (Life Technologies, with RNAse P gene used as reference gene). AlleleTyper Software (Thermo Fisher Scientific) was used to translate genetic pattern information from genotyping (Single-nucleotide polymorphisms—SNP) and copy number assay to their standardized allele name or star (*) allele nomenclature. For the NGS, the considered alleles are detailed in Table 1.

**TABLE 1:** Considered alleles.

CYP	Alleles
CYP1A2	*1K, *1F, *15, *11, *3, *16, *4, *5, *7, *6, *8
CYP2B6	*22, *10, *11, c.516G > T g.15631G > T, *4A, *16/*18, *28, *5
CYP2C9	*7, *13, *2, *27, *8, *15, *9, *10, *6, *16, *11, *3, *4, *5
CYP2C19	*17, *4, *2B, *8, *6, *9, *3, *10, *2, *7, *5
CYP2D6	*2, *29, *41, *7, *2, *9, *3, *20, *4, *14, *8, *6, *29, *17, *11, *15, *12, *10, *35, *2A
CYP3A4	*20, *3, *13, *12, *6, *2, *17, *22, *15, *1B
CYP3A5	*3/*10, *10, *2, *7, *9, *6, *3B, *8

The predicted phenotypes were based on enzyme activities of the identified alleles, as listed in the Pharmacogene Variation (PharmVar) Consortium database (Gaedigk et al., 2018; Gaedigk et al., 2019) or the PharmGKB database (Whirl-Carrillo et al., 2012). Patients were classified into poor metabolizer (PM), intermediate metabolizer (IM), normal metabolizer (NM), and ultra-rapid metabolizer (UM). For the predicted phenotype of the combined CYP3A4 and CYP3A5 genotypes, we used the classification as described by Andreu et al. PMs were defined as CYP3A4*22 carriers with the CYP3A5*3/*3 genotype, IMs were CYP3A4*22 non-carriers with the CYP3A5*3/*3 genotype or CYP3A4*22 carriers with the CYP3A5*1/*1 genotype, and NMs were CYP3A4*22 non-carriers and CYP3A5*1 carriers (Andreu et al., 2017).

### Phenotyping

Phenotyping was performed using the Geneva micrococktail which contained 50 mg caffeine, 20 mg bupropion, 10 mg flurbiprofen, 10 mg omeprazole, 10 mg dextromethorphan, 1 mg midazolam, and 25 mg fexofenadine. These probe substrates allow *in vivo* phenotyping of CYP1A2, CYP2B6, CYP2C9, CYP2C19, CYP2D6, CYP3A4/5 and P-glycoprotein, respectively (Bosilkovska et al., 2014). As previously described, the micrococktail was given orally on an empty stomach. Two hours after administration, capillary blood samples or venous blood samples were collected. In cases venous blood samples were taken, blood spots of 10 µL each were spotted on a dedicated filter card. Dried blood spots were stored at −20°C in a sealable plastic bag until analysis by a validated method using liquid chromatography tandem mass spectrometry. Phenotype determination was based on the metabolite to parent drug metabolic ratio (MR). As for genotyping, patients were classified as PM, IM, NM, and UM according to their MR (Lenoir et al., 2020).

### Clinical Data

For each patient, we collected demographic data, as well as relevant medical history and current treatments, including complementary and alternative medicine therapies. When applicable, current concomitant drugs were classified as CYP inhibitors or inducers based on our table “Interactions médicamenteuses, Cytochromes P450 et P-glycoprotéine P” (Service de pharmacologie et toxicologie cliniques and HUG, 2020), and the summary of product characteristics.

We also collected the main therapeutic classes according to ATC classification and the main reason for genotyping/phenotyping, which could be one of the following: ADR/high drug levels, inefficacy/low drug levels, DDI, International Normalized Ratio (INR) variation, prescription (preemptive testing), and other (cases not concerned by any of the other categories). Finally, based on the conclusions of the interpretive report written by a senior clinical pharmacologist, we determined whether the genotype/phenotype explained the clinical event.

### Statistical Analysis

Descriptive statistics were used. Categorical and continuous variables were described using frequency tables (n, %) and median (range), respectively. Genotype-phenotype concordance was considered adequate when the phenotype was equal to the predicted genotype based on the identified alleles. All analyses were performed using the SPSS® software package, version 25 (IBM corporation, Armonk, NY, United States).

## Results

### Population Characteristics

**FIGURE 1:**
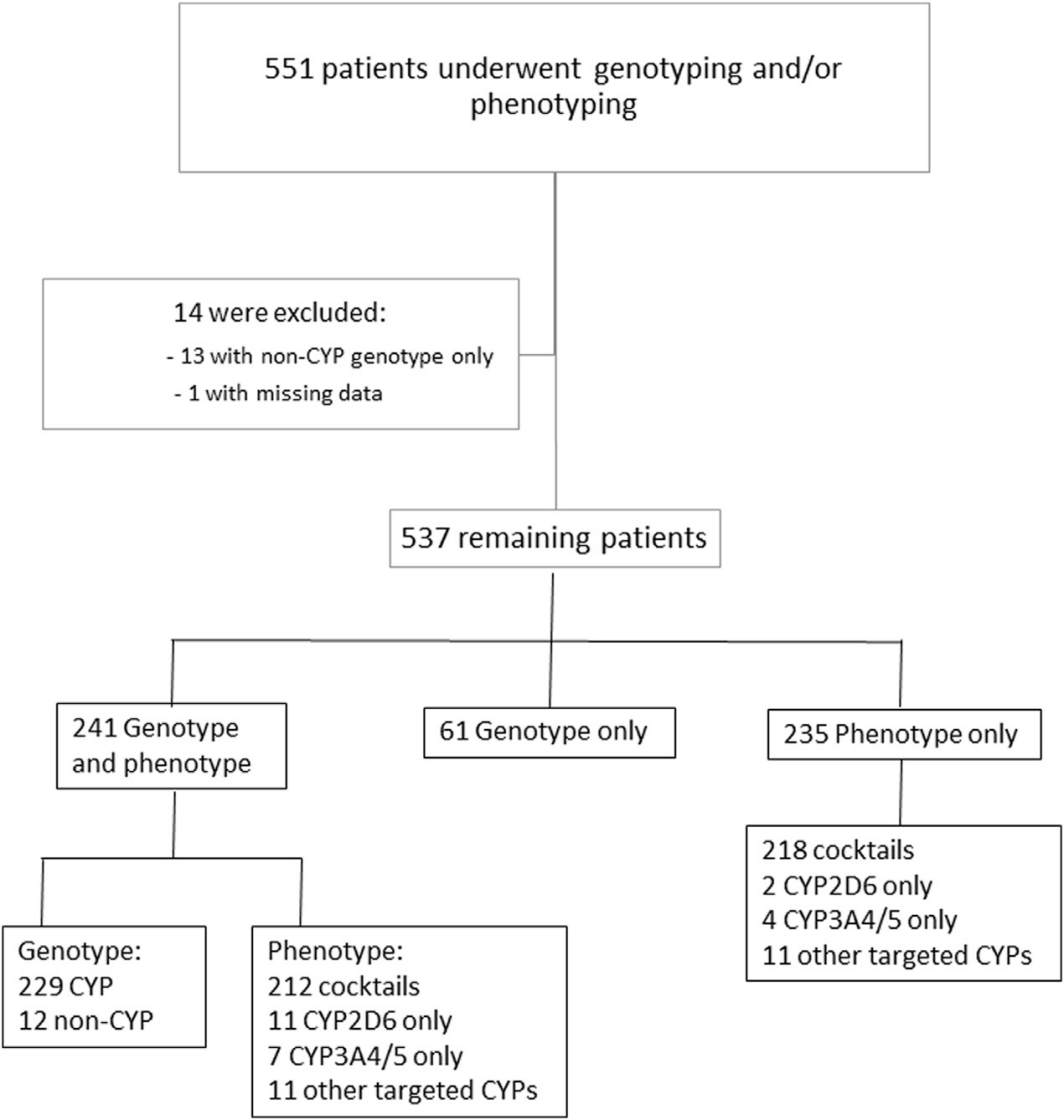
Study flowchart.

Between January 2014 and December 2020, a total of 551 patients underwent genotyping and/or phenotyping tests. Among them, 13 patients had only non-CYP450 genotyping (ABCB1, COMT, SLCO1B1, and OPRM1) and one had missing data; these patients were therefore excluded from the analysis (Figure 1). The mean age of the 537 remaining patients was 48.6 years old (range: one month-90 years) and 52.3% (*n* = 281) were women. Among the 537 patients, 241 (45%) underwent simultaneous genotyping and phenotyping, 235 (44%) underwent phenotyping only, and 61 (11%) underwent genotyping only (Figure 1).

### Genotype and Phenotype

**FIGURE 2:**
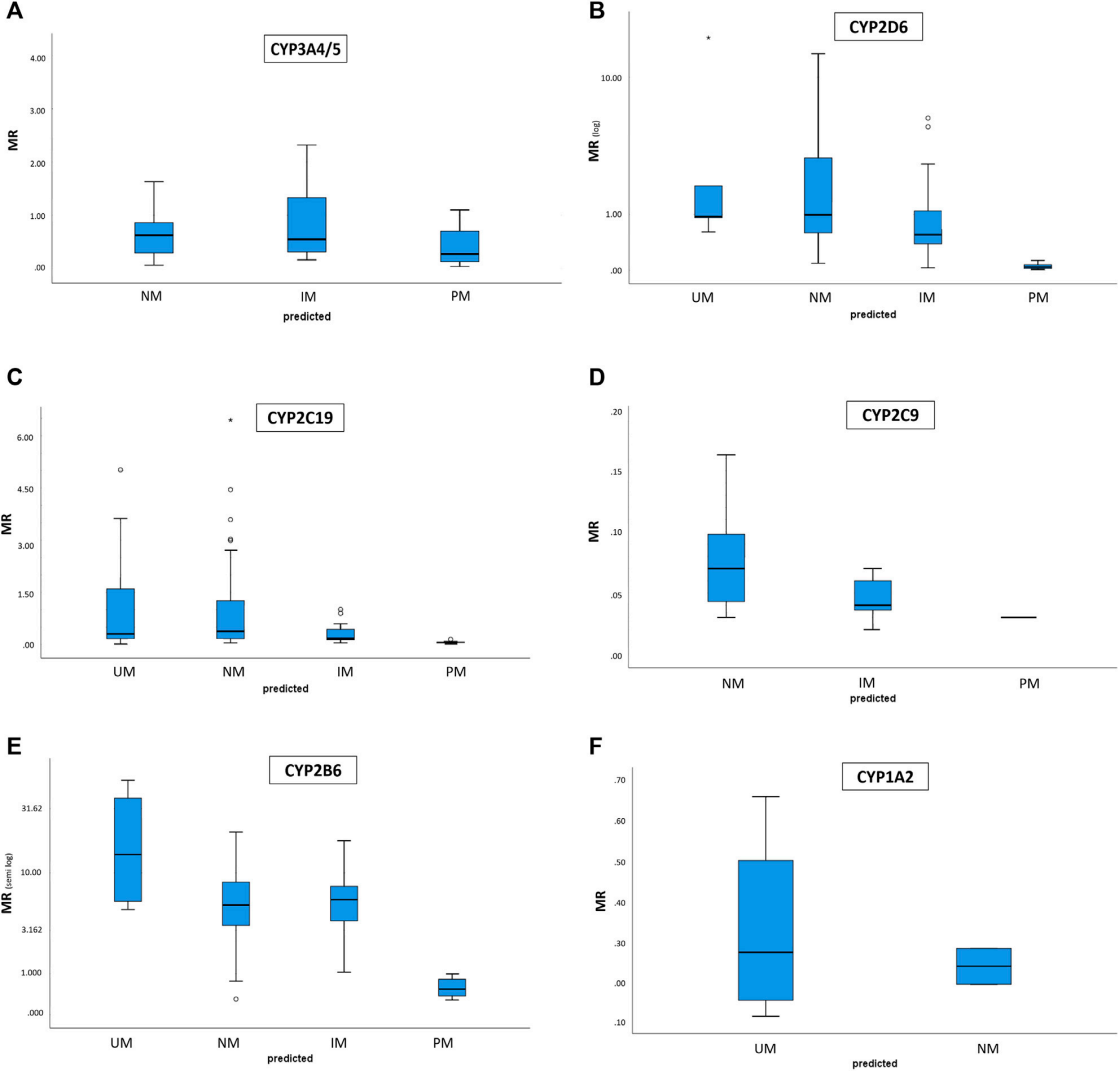
Distribution of metabolic ratios (y axis) for each phenotype predicted by the genotype (x axis). Boxes indicate the interquartile ranges. **(A)** CYP1A2. **(B)** CYP2B6. **(C)** CYP2C9. **(D)** CYP2C19. **(E)** CYP2D6. **(F)** CYP3A4 and CYP3A5.

A total of 241 patients underwent simultaneous genotyping and phenotyping, allowing for genotype-phenotype concordance assessment. The majority of patients received the Geneva microcktail (*n* = 212) while the other patients (*n* = 29) had targeted phenotyping, i.e. focusing on one, two, or eventually three CYP enzymes, usually in children to minimize the exposure to non-authorized substances in this population, or because targeted phenotyping was justified by the clinical context. The predicted phenotypes according to the identified alleles are presented in Table 2. Figure 2 shows the distribution of metabolic ratios for each phenotype predicted by the genotype. The concordance between the predicted and measured phenotypes is presented in Table 3. As illustrated in Figure 2 and Table 3, genotyping allowed correct prediction of PM phenotypes for most of the studied CYPs isoenzymes, with a 100% concordance between the predicted and measured phenotype for CYP2C9, CYP2C19, and CYP2D6 PM. On the other hand, concordance rates between the predicted and measured phenotype for IM, NM, and UM, whatever the considered isoenzyme, varied widely with concordance rates ranging from 19 to 100%. CYP2C9 phenotypic IM and NM were correctly predicted by the genotype in approximately 60% of the cases. CYP2C19 and CYP2D6 phenotypic IM were frequently well predicted by the genotype (concordance in 91 and 73% of the cases respectively) whereas the opposite was true for CYP2C19, CYP2D6, and CYP3A4/5 NM, in which a concordance between the predicted and measured phenotype was observed in 30–38% of cases only. Finally, except for CYP2B6, individuals with a UM genotype frequently displayed a discordant phenotype (63% of cases for CYP1A2, and 80% of cases for CYP2C19 and CYP2D6). In these cases of discordance, a phenotypic switch due to the concomitant use of a CYP inhibitor explained 14–47% of cases.

**TABLE 2:** In patients with simultaneous genotyping and phenotyping, CYP predicted phenotype based on the identified alleles.

	—	PM	IM	NM	UM	Unknown
**CYP1A2**	Number of individuals (%)	0	0	2 (17%)	9 (75%)	1 (8%)
	Genotypes	—	—	*1B/*1B	*1F/*1F, *1/*1F, *1A/*1F, *1B/*1F	*1N/*1N
**CYP2B6**	Number of individuals (%)	6 (16%)	10 (26%)	17 (45%)	4 (11%)	1 (2%)
	Genotypes	*6/*6, *9/*9	*1/*6, *1/*9, *4/*6, *5/*6	*1/*1, *1/*2, *1/*5, *2/*2B	*1/*22, *2/*22, *5/*22	*5/*10
**CYP2C9**	Number of individuals (%)	2 (3%)	20 (32%)	40 (65%)	NA	0
	Genotypes	*2/*3	*1/*2, *1/*3	*1/*1, *1/*9	NA	—
**CYP2C19**	Number of individuals (%)	7 (6.7%)	23 (21.9%)	48 (45.7%)	27 (25.7%)	0
	Genotypes	*2/*2	*1/*2, *1/*3, *2/*17	*1/*1	*17/*17, *1/*17	—
**CYP2D6**	Number of individuals (%)	11 (8.1%)	52 (38.5%)	66 (48.9%)	5 (3.7%)	1 (0.8%)
	Genotypes	*3/*4, *4/*4, *5/*5, *6/*6	*1/*3, *1/*4, *1/*5, *2A/*4, *4/*35, *5/*41	*1/*1, *1/*2, *1/*2A, *1/*9, *1/*10, *1/*41	Functional allele xN	*41/*119
**CYP3A4-CYP3A5**	Number of individuals (%)	8 (11%)	52 (73%)	10 (14%)	NA	1 (2%)
	Genotypes	*1/*22–*3/*3, *22/*22–*3/*3	*1/*1–*3/*3	*1/*1–*1/*1, *1/*1-*1/*3, *1/*1B–*1/*3	NA	*1/*1–*4/*4

PM: poor metabolizer; IM: intermediate metabolizer; NM: normal metabolizer; UM: ultra-rapid metabolizer. CYP: cytochrome P450. NA: not applicable. For CYP2D6 and CYP3A4-CYP3A5, only the principal identified genotypes are given.

**TABLE 3:** Concordance between the predicted and measured and phenotypes, given as n (%).

	—	PM	IM	NM	UM	Phenotypic switch^a^
**CYP1A2**	Concordant	—	—	2 (100%)	3 (37%)	—
	Non concordant	—	—	0	5 (63%)	2 (40%) (estradiol, paroxetine)
**CYP2B6**	Concordant	4 (37%)	0	6 (38%)	4 (100%)	—
	Non concordant	2 (33%)	10 (100%)	10 (62%)	0	6 (27%) (isavuconazole, cyclophosphamide)
**CYP2C9**	Concordant	2 (100%)	10 (59%)	25 (66%)	NA	—
	Non concordant	0	7 (41%)	13 (34%)	NA	1 (5%) (sulfamethoxazole)
**CYP2C19**	Concordant	7 (100%)	20 (91%)	14 (33%)	5 (19%)	—
	Non concordant	0	2 (9%)	28 (67%)	21 (81%)	24 (47%) (es)omeprazole, fluconazole, voriconazole
**CYP2D6**	Concordant	11 (100%)	36 (73%)	25 (38%)	1 (20%)	—
	Non concordant	0	13 (27%)	40 (62%)	4 (80%)	18 (32%) [paroxetine, venlafaxine, (es)citalopram, duloxetine, sertraline, quetiapine, risperidone]
**CYP3A4/5**	Concordant	5 (63%)	24 (48%)	3 (30%)	NA	—
	Non concordant	3 (37%)	26 (52%)	7 (70%)	NA	5 (14%) (azole antifungal)

PM: poor metabolizer; IM: intermediate metabolizer; NM: normal metabolizer; UM: ultra-rapid metabolizer. CYP: cytochrome P450. NA: not applicable

a Number of cases with a phenotypic switch explaining the non-concordance (with examples of involved comedications)

As illustrated in Figure 2, for CYP1A2, carriers and non-carriers of the CYP1A2*1F allele, which has been associated with increased inducibility (Thorn et al., 2012), had great overlap in their MR. For CYP2B6, patients predicted as NM et IM according to their genotype also displayed great overlap in their MR. This wide distribution of MR associated with overlap between phenotype subgroups was also observed for CYP2C19 genotypic UM and NM, CYP2D6 genotypic UM and NM, and CYP3A genotypic NM and IM.

### Phenotype

A total of 235 patients underwent phenotyping only. The majority of patients received the Geneva microcktail (*n* = 218) while the other patients (*n* = 17) had targeted phenotyping for the reasons explained previously. The distribution of the phenotypes, according to CYP isoenzymes is presented in Table 4. As expected, the majority of patients were categorized as NM, regardless of the isoenzyme examined. The proportion of patients displaying slow CYP metabolism (PM + IM) was low for CYP2B6 (approximately 2%), intermediate for CYP1A2 (10%), CYP2C9 (18%), and CYP3A (17%), and high for CYP2C19 and CYP2D6 (more than 30%). Finally, the proportion of patients categorized as UM was quite high for CYP1A2 (24%) and CYP2B6 (15%), while it was measured around 7–11% for the other isoenzymes (CYP2C9, CYP2C19, CYP2D6, and CYP3A).

**TABLE 4:** Distribution of measured phenotypes.

	—	PM	IM	NM	UM
**CYP1A2**	Number of individuals (%)	23 (10.5%)	0	145 (65.9%)	52 (23.6%)
	Mean metabolic ratio (SD)	0.15 (0.41)	NA	0.34 (0.1)	0.8 (0.29)
**CYP2B6**	Number of individuals (%)	1 (0.5%)	2 (0.9%)	181 (83.8%)	32 (14.8%)
	Mean metabolic ratio (SD)	0.39 (NA)	0.56 (0)	2.21 (1.1)	9.11 (7.1)
**CYP2C9**	Number of individuals (%)	26 (12.0%)	14 (6.5%)	161 (74.5%)	15 (6.9%)
	Mean metabolic ratio (SD)	0.021 (0.01)	0.039 (0.001)	0.066 (0.018)	0.14 (0.021)
**CYP2C19**	Number of individuals (%)	43 (20.3%)	23 (10.8%)	127 (59.9%)	19 (9.0%)
	Mean metabolic ratio (SD)	0.15 (0.07)	0.34 (0.069)	1.12 (0.59)	3.45 (1.3)
**CYP2D6**	Number of individuals (%)	18 (8.0%)	62 (27.7%)	128 (57.1%)	16 (7.1%)
	Mean metabolic ratio (SD)	0.08 (0.07)	0.49 (0.19)	1.78 (0.97)	8.78 (3.54)
**CYP3A4-CYP3A5**	Number of individuals (%)	33 (14.5%)	5 (2.2%)	164 (71.9%)	26 (11.4%)
	Mean metabolic ratio (SD)	0.17 (0.08)	0.3 (0.022)	0.74 (0.38)	3.06 (0.86)

PM: poor metabolizer; IM: intermediate metabolizer; NM: normal metabolizer; UM: ultra-rapid metabolizer. CYP: cytochrome P450. NA: not applicable. SD: standard deviation

### Genotype

Sixty-one patients underwent genotyping only, with a total of 115 genes evaluated. The most frequently investigated enzyme was CYP2D6 (*n* = 33), followed by CYP3A4 and CYP3A5 (*n* = 23 for both). Other genes were infrequently (CYP2C19 *n* = 16; CYP2C9 *n* = 10; CYP2B6 *n* = 8) or very rarely (CYP1A2 *n* = 2) investigated.

### Association Between Clinical Response and Genotype And/Or Phenotype

**FIGURE 3:**
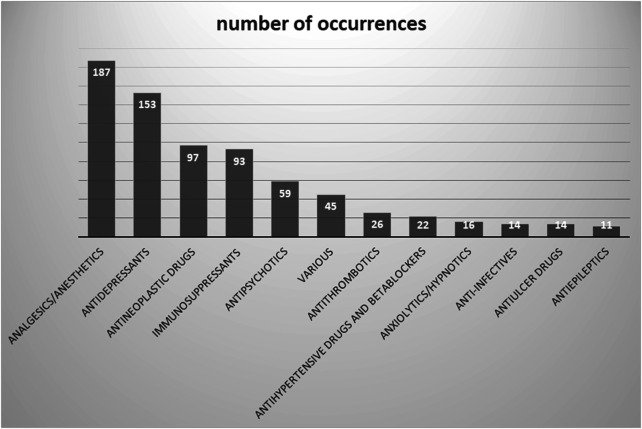
Involved therapeutic classes.

The majority of clinical situations were observed with analgesic/anesthetic drugs (*n* = 187), followed by antidepressants (*n* = 153), antineoplastics (*n* = 97), and immunosuppressants (*n* = 93) (Figure 3).

Genotypic and/or phenotypic explorations were mainly performed because of inefficacy/low drug levels (38%) followed by ADR/high drug levels (33%), and preemptively before prescribing (18%). Based on the clinical report, the genotype and/or phenotype results interestingly explained or at least contributed to almost half of the clinical event (44% of the cases). In 26% of the cases, the association between the genotype/phenotype and a clinical event could not be evaluated (preemptive testing, evaluation of a DDI, INR variation) (Table 5). In the 237 cases where the genotype/phenotype could contribute to the clinical event, the differential contribution of the different tests were as followed. In 47% of the patients who underwent genotyping only, the results explained or contributed to the clinical event. This proportion was 34% in patients who underwent phenotyping only, and 53% in patients who underwent simultaneous genotyping and phenotyping. In most of the patients in whom the genotype provided an explanation, the clinical event was ADR/high drug levels and the most frequently involved therapeutic classes were psychotropic and analgesic/anesthetic drugs. In most of the patients in whom the phenotype provided an explanation, the clinical event was of inefficacy/low drug levels, with also psychotropic and analgesic/anesthetic drugs frequently involved. Finally, combined genotype and phenotype mostly explained ADR/high drug levels with various therapeutic classes involved (psychotropic, analgesic, antineoplastic, immunosuppressant, and antithrombotic drugs).

**TABLE 5:** Characteristics of patients and reasons for genotyping/phenotyping.

	Number	%
Gender (*n* total = 537)	—	—
Female	281	52.3
Male	256	47.7
Reason for genotyping/phenotyping (*n* total = 546^a^)	—	—
inefficacy/low drug levels	208	38.1
ADR/high drug levels	178	32.6
prescription (preemptive testing)	100	18.3
DDI	16	2.9
INR variation	11	2.0
other	33	6.0
Clinical event explained by genotype/phenotype	—	—
Yes	237	44.1
No	161	30.0
Not applicable	139	25.9

a Some patients had two reasons for genotyping/phenotyping

ADR: adverse drug reaction, DDI: drug-drug interaction, INR: international normalized ratio

## Discussion

Our retrospective study describes for the first time the clinical use of the Geneva CYP450 phenotyping micrococktail in patients suffering from various comorbidities and taking numerous comedications. The new version of the Geneva phenotyping cocktail using low doses of probe substrates was validated in 2014 in healthy volunteers (Bosilkovska et al., 2014) using CYP inhibitors and inducers to generate MR thresholds allowing to characterize the different phenotypes (e.g. PM, NM, UM). We report here concordance between the genotypes and the measured phenotypes as well as associated MR. We showed that the concordance between the predicted and measured phenotypes was excellent for PM of all CYPs. Genotypic prediction of CYP2C9, CYP2C19, and CYP2D6 IM was satisfying to good, and prediction performance of CYPs NM was moderate. On the other hand, discrepancies were frequent for patients predicted as UM. Discrepancies between the predicted phenotype as based on genotyping and the measured phenotyping were sometimes explained by concurrent medication (phenotypic switch). A previous article from our research group showed that the overall concordance rate between the predicted and measured phenotype was around 50% for CYP2D6 and CYP2C9 using a previous version of the Geneva cocktail (Rollason et al., 2020b). The poor correlation between genotype and phenotype for UM had already been shown previously for CYP2D6 using the AmpliChip CYP450 test for genotyping and dextromethorphan/dextrorphan urine metabolic ratio for phenotyping (Rebsamen et al., 2009). More recently, Dorado et al. also showed that CYP2D6 genotype was not a good predictor of UM phenotype as measured with debrisoquin probe drug. Only 25% of phenotypic UM were explained by their genotype (carrying more than two active CYP2D6 genes) (Dorado et al., 2017). De Andrés et al. evaluated the correlation between CYPs genotype and phenotype using a cocktail approach in several populations (De Andrés et al., 2016; de Andrés et al., 2017; de Andrés et al., 2020). Their different studies showed that the drug-metabolizing capacity predicted from the genotype was frequently not concordant with the actual capacity as measured by phenotyping. For example, in their study in Mexican Amerindian, CYP2C19 genotypic UM displayed a wide range of MR, and several CYP2C19 genotypic NM had higher metabolic activity than UM. Similar to our results, they observed no association between CYP1A2*1F and enhanced CYP1A2 metabolic capacity (de Andrés et al., 2017).

Our results and those from others underscore the complementary roles of genotyping and phenotyping tests when aiming to personalize patient treatment. To minimize the risk of ADRs or therapeutic failure, the individual’s drug metabolic capacities should be assessed at the time of initiation of treatment, for drugs metabolized by CYPs. Since such preemptive testing is time-consuming and might be difficult to implement in clinical practice, the simpler approach of genotyping has been suggested. Indeed, since the genome is constant throughout a lifetime, this might in theory be a useful surrogate marker for drug toxicity or inefficacy (Waring, 2020). However, prediction from the genotype can be limited by the lack of accurate results for unknown genotypes/variants (particularly with NGS technologies) and misclassification of phenotype due to errors in genetic predictions. Moreover, measured phenotype can be influenced by environmental factors such as concomitant drugs, dietary habits, or concomitant diseases (McGraw et al., 2018).

The complex interplay between genetics and drug-drug interaction has been recently reviewed by Storelli et al. Through a systematic review of case reports, they identified several mechanisms of complex gene-drug interaction such as enhancement of the magnitude of interaction due to a genetic variant directly impacting the CYP isoform of interest, increased vulnerability to phenoconversion caused by a genetic variant directly affecting the inhibited/induced metabolic pathway, increased exposure of the perpetrator drug due to genetic polymorphisms, and modification of the relative contribution of a minor pathway by a genetic variant affecting the major pathway. For example, a PM of a specific CYP isoform will not be greatly affected by an inhibitor of this isoform. In healthy CYP2D6 PM Caucasian volunteers, no increase in the AUC of metoprolol was observed when coadministered with dronedarone, a moderate CYP2D6 inhibitor (Storelli et al., 2018). In other words, CYP2D6 PMs are not sensitive to phenoconversion or phenotypic switch by CYP2D6 inhibitors. Phenoconversion corresponds to the modification of the activity of a drug-metabolizing enzyme by an inhibitor/inducer that mimics a genetic defect. For example, a CYP2D6 genotypic EM patient undergoing phenoconversion with a CYP2D6 inhibitor would behave pharmacologically as a PM. Other causes of phenoconversion include liver transplant or liver disease (Shah and Smith, 2015) as well as possibly other diseases (inflammation, surgery, etc.).

From a clinical point of view, the genotypes and/or phenotypes contributed to the observed clinical event (i.e. inefficacy, ADR) in 44% of the cases globally. When looking in more detail into the differential contribution of genotyping versus phenotyping results, we observed that simultaneous genotyping and phenotyping allowed explaining the clinical event in a greater proportion of patients then when doing genotyping or phenotyping only. This 44% proportion is quite similar to previously published results from our group focusing on analgesic (Rollason et al., 2020a) and psychotropic drugs (Lloret-Linares et al., 2017), with data collected until the end of 2014. As in the previous analysis, the main therapeutic classes in our study were analgesic/anesthetic drugs and antidepressants. A possible bias in this observation is the other clinical specialty of clinical pharmacologists in our division (experts in pain and psychopharmacology). On the other hand, many drugs from these two therapeutic classes are also subject to metabolism by CYP450, therefore potentially influenced by CYP genetic polymorphisms. This is reflected by numerous genotype-based dosing recommendations found on the PharmaGKB website (https://www.pharmgkb.org/).

When classifying patients in metabolic subgroups according to their genotypes, there were around 8% of CYP2D6 PM, 39% of IM, 49% of NM, and 4% of UM. Geneva is considered to have a population from various ethnicities. Our frequencies of extreme metabolizer (PM and UM) were similar to those reported by del Tredici et al. in their study conducted in more than 100’000 patients in the US from multiple ethnic groups (6% PM and 2% UM), while IM were more prevalent in our study (39 versus 11%) (Del Tredici et al., 2018). As reported by Gaedick et al., the distribution frequency of CYP2D6 shows considerable differences across different world populations (Gaedigk et al., 2017). Regarding CYP2C19, we found 7% of PM, 20% of IM, 45% of NM, and 25% of rapid/ultrarapid metabolizers. Our frequency of PM was higher than what is usually observed in Caucasians from different parts of the world as reported by Fricke-Galindo et al. (2–3% approximately), but the frequency of UM was comparable (Fricke-Galindo et al., 2016). The frequency of CYP2C19 PM was also much higher in our study than reported by Fricke-Galindo et al. for Europe (20.3 versus 2.2% (Fricke-Galindo et al., 2016).

Our study has some limitations. Bias related to retrospective analysis might have led to missing information, such as follow-up data if the genotyping and/or phenotyping results led to dosage/therapeutic changes. For some isoenzymes such as CYP1A2 and CYP2B6, the limited number of patients undergoing simultaneous genotyping and phenotyping limited definitive conclusions on genotype/phenotype concordance.

## Conclusion

Our study reported for the first time the clinical use of the Geneva micrococktail in patients as well as genotype/phenotype concordance. We showed that genotyping and/or phenotyping tests were useful in explaining or solving clinical events in almost half of the cases. We also observed that genotype/phenotype concordance was excellent for poor metabolizers but more variable for normal, and ultrarapid metabolizers. Our results highlight the complementary aspects of genotyping and phenotyping tests in helping to individualize drug therapy, and these tests should therefore be offered concomitantly more routinely in the clinic.

## Data Availability

The raw data supporting the conclusion of this article will be made available by the authors, without undue reservation.
